# Fibronectin Protects from Excessive Liver Fibrosis by Modulating the Availability of and Responsiveness of Stellate Cells to Active TGF-β

**DOI:** 10.1371/journal.pone.0028181

**Published:** 2011-11-28

**Authors:** Nina Kawelke, Matthaeus Vasel, Carla Sens, Anja von Au, Steven Dooley, Inaam A. Nakchbandi

**Affiliations:** 1 Translational Medicine, Max-Planck Institute for Biochemistry, Martinsried, Germany; 2 Institute for Immunology, University of Heidelberg, Heidelberg, Germany; 3 Department of Medicine II, University of Heidelberg at Mannheim, Mannheim, Germany; University of Pittsburgh, United States of America

## Abstract

Fibrotic tissue in the liver is mainly composed of collagen. Fibronectin, which is also present in fibrotic matrices, is required for collagen matrix assembly in vitro. It also modulates the amount of growth factors and their release from the matrix. We therefore examined the effects of the absence of fibronectin on the development of fibrosis in mice.

Conditional deletion of fibronectin in the liver using the Mx promoter to drive cre expression resulted in increased collagen production and hence a more pronounced fibrosis in response to dimethylnitrosamine in mice. Exclusive deletion of fibronectin in hepatocytes or normalization of circulating fibronectin in Mx-cKO mice did not affect the development of fibrosis suggesting a role for fibronectin production by other liver cell types. The boosted fibrosis in fibronectin-deficient mice was associated with enhanced stellate cell activation and proliferation, elevated concentrations of active TGF-β, and increased TGF-β-mediated signaling.

In vitro experiments revealed that collagen-type-I production by fibronectin-deficient hepatic stellate cells stimulated with TGF-β was more pronounced, and was associated with augmented Smad3-mediated signaling. Interfering with TGF-β signaling using SB431542 normalized collagen-type-I production in fibronectin-deficient hepatic stellate cells. Furthermore, precoating culture plates with fibronectin, but not collagen, or providing fibronectin fibrils unable to interact with RGD binding integrins via the RGD domain significantly diminished the amount of active TGF-β in fibronectin-deficient stellate cells and normalized collagen-type-I production in response to TGF-β stimulation. Thus, excessive stellate cell activation and production of collagen results from increased active TGF-β and TGF-β signaling in the absence of fibronectin.

In conclusion, our data indicate that fibronectin controls the availability of active TGF-β in the injured liver, which impacts the severity of the resulting fibrosis. We therefore propose a novel role for locally produced fibronectin in protecting the liver from an excessive TGF-β-mediated response.

## Introduction

The development of liver fibrosis is one of the early steps in the pathogenesis of advanced liver disease and liver failure. Hepatic stellate cells play a key role in this process by producing extracellular matrix molecules that become incorporated in a distorted fibrogenous network hindering normal function in hepatocytes [1]. Transforming-growth-factor-β (TGF-β) has been described as a major stimulator of stellate cell activation, and hence, extracellular matrix production [2].

Fibronectin is one of the molecules produced by hepatic stellate cells [3]. It is also part of the extracellular matrix [4]. Several isoforms have been described and many functions have been attributed to fibronectin. Fibronectin is important for the assembly of a collagen matrix *in vitro* [5], [6]. Its continuous presence also supports matrix integrity, both *in vitro* and *in vivo* [5], [7]. It further regulates cell proliferation and cell cycle progression [8]. We have shown that two isoforms of fibronectin reflect the severity of liver fibrosis in patients with chronic hepatitis C raising the possibility that fibronectin itself may play a role in the pathogenesis of fibrosis [9].

In order to define the role of fibronectin in the liver on the development of liver fibrosis we undertook the following study in which fibronectin was conditionally deleted in various cell types in mice. Liver injury was induced using dimethylnitrosamine (DMN) and experiments on liver tissue from these mice were complemented with *in vitro* experiments in isolated stellate cells. Our results show that deletion of fibronectin leads to an increase in stellate cell activation, both at baseline and after stimulation with TGF-β. This is due to an increase in TGF-β bioavailability and results in a more pronounced fibrosis.

## Results

### Deletion of fibronectin in hepatic stellate cells, hepatocytes, Kupffer cells and endothelial cells

**Figure 1:**
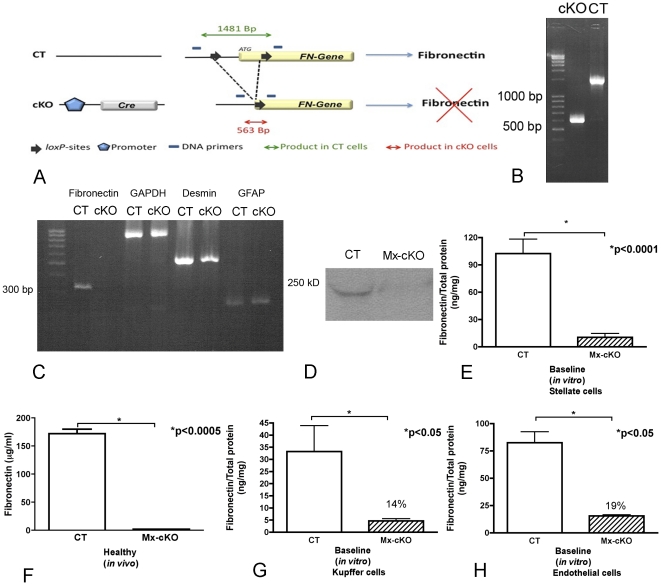
Evidence for successful deletion of fibronectin (FN) in vitro and in mice in vivo. Stellate, Kupffer, and endothelial cells were isolated from Mx-conditional knockout (cKO) mice and control (CT) littermates. Stellate cells were cultured for 24 hours on plastic in the presence of 10% fibronectin-depleted fetal calf serum (FCS) (Fibronectin concentration in FCS after depletion equaled 8 ng/ml) and then collected. (**A**) A simplified map of fibronectin DNA. Shown are the locations of the *loxP* sites around exon 1 (including the ATG sequence) and the sites of the primers used for DNA characterization shown in 1B. The fibronectin gene is made nonfunctional by loss of the DNA between the *loxP* sites in the presence of cre. (**B**) PCR analysis of genomic DNA shows successful deletion of the fibronectin fragment containing the ATG sequence in cKO stellate cells. (**C**) mRNA expression by reverse transcription PCR shows that fibronectin expression is absent in cKO stellate cells compared to CT in the presence of almost equivalent expression of desmin and GFAP, which are markers for stellate cells, as well as the house keeping gene GAPDH. (**D**) Immune precipitation of stellate cell lysates from CT and cKO cells followed by Western blot for fibronectin shows almost absent fibronectin production in cKO stellate cells after 4 days in culture. (**E**) ELISA for fibronectin in conditioned media collected from the Mx-cKO stellate cells shows lower levels of fibronectin compared to conditioned media of littermate CT cells. Values were corrected to total protein measured by BCA. (**F**) Deletion of fibronectin *in vivo* using the Mx-promoter results in a significant decrease in circulating fibronectin, because the Mx-promoter also deletes fibronectin in hepatocytes, which produce most of circulating fibronectin. N = 7 and 6. (**G**) ELISA for fibronectin in conditioned media collected from the Mx-cKO Kupffer cells shows lower levels of fibronectin (14% of CT) compared to conditioned media of littermate CT cells. Values were corrected to total protein measured by BCA. (**H**) ELISA for fibronectin in conditioned media collected from the Mx-cKO endothelial cells shows lower levels of fibronectin (19% of CT) compared to conditioned media of littermate CT cells. Values were corrected to total protein measured by BCA.

Hepatic stellate cells were isolated from control mice (CT) and conditional knockout mice carrying the Mx promoter attached to cre recombinase (Mx-cKO). All mice were homozygote for the floxed fibronectin gene. The Mx promoter was activated *in vivo* in Mx-cKO mice and through the resulting expression of the attached cre enzyme the homozygote fibronectin floxed genes present in the cells were deleted (Figures 1A and 1B) [10]. 97±2% of the isolated hepatic stellate cells stained for either desmin or glial fibrillary acidic protein (GFAP), both of which are markers used for identifying stellate cells [11] suggesting a high purity. Deletion of fibronectin was confirmed at the DNA (Figures 1A and 1B), mRNA (Figure 1C) and protein level (Figure 1D: Western blot; Figure 1E: ELISA of conditioned media) in cells and conditioned media. Thus, activation of the Mx promoter successfully deleted fibronectin in hepatic stellate cells. In addition to deleting fibronectin in stellate cells, the activated Mx promoter can efficiently delete fibronectin in hepatocytes, which are responsible for the production of circulating fibronectin. Therefore, loss of fibronectin in hepatocytes in mice that express Mx-cre and are homozygote for floxed fibronectin can be easily confirmed by measuring circulating plasma fibronectin, which in this case confirmed successful deletion (Figure 1F). Other cell types that can be affected by the activation of the Mx promoter are Kupffer and endothelial cells. The identity of isolated Kupffer cells was confirmed by staining with F4/80 and absence of staining with desmin or GFAP (data not shown). ELISA of conditioned media confirmed deletion of fibronectin to 14% of control values (Figure 1G). The identity of isolated endothelial cells was confirmed by positive staining with acetylated-LDL and absence of staining with F4/80, desmin and GFAP (Data not shown). ELISA of conditioned media also showed a decrease in fibronectin protein to 19% of control values (Figure 1H).

### Induction of liver fibrosis

**Figure 2:**
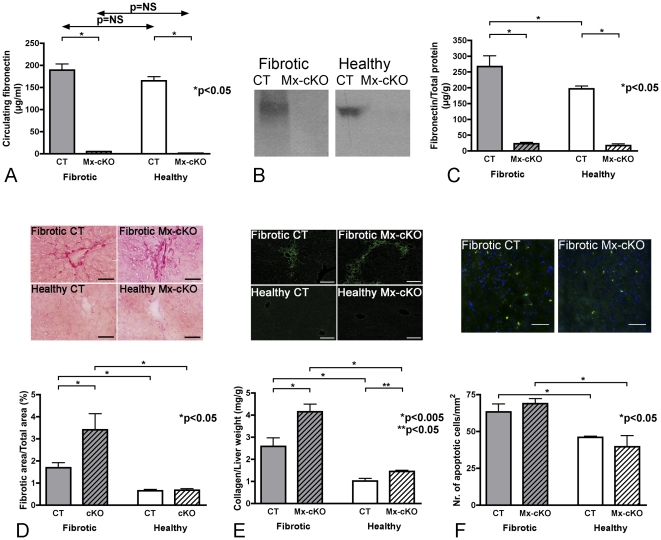
Induction of liver fibrosis. Fibrosis was induced in control mice (CT) and in conditional knockout mice in which fibronectin was deleted upon activation of the Mx promoter (Mx-cKO) using dimethylnitrosamine (DMN), and healthy littermate CT and cKO mice were treated with normal saline (NaCl 0.9%) instead. (**A**) ELISA of circulating fibronectin to confirm deletion in Mx-conditional knockout mice (Mx-cKO). N = 16 per group. (**B**) Representative Western blot for fibronectin in liver tissue from fibrotic and healthy mice showing the absence of a signal for fibronectin in Mx-cKO mice. Equal protein amounts as determined by BCA measurements were loaded on the gel. (**C**) Fibronectin ELISA of liver lysates showing an increase in fibronectin in fibrotic CT mice compared to healthy CT mice but no significant increase in fibrotic Mx-cKO mice compared to healthy Mx-cKO mice. (**D**) Upper panel: Sirius-red staining of the extracellular matrix suggesting an increase in matrix accumulation in fibrotic Mx-cKO mice. *Bars represent 50* *µm.* Lower panel: Morphometric analysis of the fibrotic area on three sections per mouse. N = 6–9 mice/group. (**E**) Upper panel: Collagen-type-I staining by immunofluorescence showing an increase in staining in fibrotic Mx-cKO mice. *Bars represent 100* *µm.* Lower panel: Collagen (determined by measuring hydroxyproline) is increased in livers of fibrotic Mx-cKO mice (1.6-fold increase in fibrotic Mx-cKO mice compared to fibrotic controls). Of note, healthy Mx-cKO mice already show a significant increase in collagen, when compared to healthy CT (1.3-fold increase), albeit at lower amounts than fibrotic mice. Triplicate measurements were performed/mouse. N = 5–14 mice/group. (**F**) No difference in the total number of apoptotic cells is found between CT mice and Mx-cKO mice, as determined by TUNEL staining to identify apoptotic cells (in green) and nuclei (DAPI in blue). Upper panel: Examples of areas that were TUNEL stained in fibrotic CT and Mx-cKO mice. *Bars represent 200* *µm.* Lower panel: Quantification of the number of apoptotic cells. 3 sections/mouse were analyzed. N = 3 mice/group.

We next induced liver fibrosis in cKO mice in which the Mx promoter was activated (Mx-cKO) and control (CT) mice using dimethylnitrosamine (DMN). ELISA of circulating fibronectin confirmed successful deletion (Figure 2A) [12], [13]. To exclude the possibility that some fibronectin was generated in fibrotic livers by intronic or alternate promoters we performed quantitative RT-PCR for mRNA encoding the C-terminal part of fibronectin, the EDA, or the EDB domain. There was more than 90% decrease in all three products in fibrotic Mx-cKO mice compared to CT mice (Figure S1). Fibronectin content was examined in the liver by Western blotting (Figure 2B), which showed a difference in fibronectin amounts between fibrotic CT and fibrotic Mx-cKO. This was further confirmed by ELISA of liver lysates (Figure 2C). No significant increase in the amount of fibronectin was seen after induction of liver fibrosis in cKO animals compared to healthy cKO mice however, further supporting the conclusion that fibronectin deletion in the liver was successful *in vivo.*

### Effect on extracellular matrix accumulation

DMN treatment resulted in increased sirius-red staining (i.e., extracellular matrix) in fibrotic-Mx-cKO mice when compared to fibrotic CT mice (Figure 2D). Further, collagen-type-I staining suggested that more collagen accumulated in fibrotic Mx-cKO mice (Figure 2E-upper panel). This was confirmed using a biochemical method measuring the amount of hydroxyproline, which is a component and hence a marker for collagen, in liver lysates (Figure 2E-lower panel). Using this method, healthy Mx-cKO showed an increase in collagen (1.3-fold) compared to healthy controls (p<0.05), while treatment with DMN resulted in a larger increase in collagen-type-I in fibrotic-Mx-cKO (1.6-fold) compared to fibrotic-CT (p<0.05).

### Role of apoptosis

The development of liver fibrosis in response to DMN is preceded by increased cell death leading to signals that promote extracellular matrix accumulation [14], [15]. Fibronectin may exert antiapoptotic effects [15], [16]. We therefore sought to determine whether loss of fibronectin in Mx-cKO mice is associated with increased apoptosis and hence increased fibrosis [14], [15]. No difference in the total number of apoptotic cells (stained with TUNEL or with cleaved caspase 3 (ASP175)) between fibrotic Mx-cKO and fibrotic CT livers could be detected (Figure 2F and data not shown). Similarly, we could not detect any differences in the levels of the liver enzymes AST or ALT that reflect hepatocyte necrosis (Figure S2). Thus the increase in matrix accumulation in Mx-cKO mice is not due to increased apoptosis and hence it was not due to loss of an antiapoptotic effect of fibronectin in the liver.

### Defining the cell type associated with increased matrix accumulation in Mx-cKO mice

#### Hepatocytes

**Figure 3:**
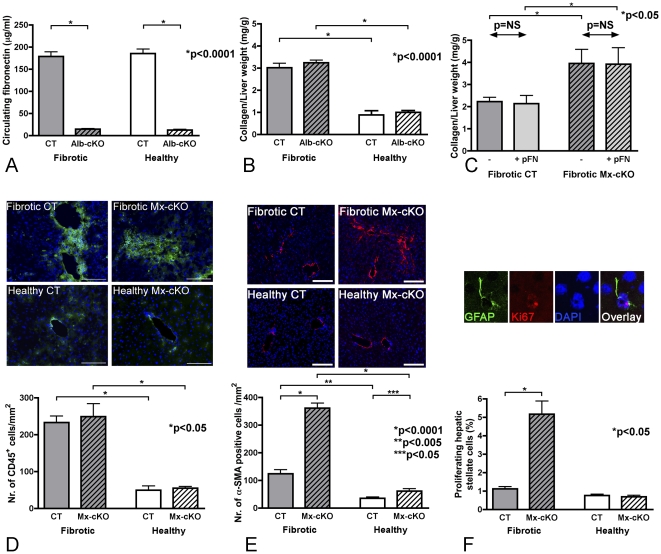
Determining the cell type associated with increased collagen production in Mx-cKO mice. Experiments shown in panels A and B were performed in Alb-cKO mice lacking fibronectin in the hepatocytes only in order to determine whether the absence of fibronectin in hepatocytes affects the degree of fibrosis. Experiments shown in panels C–F were performed in Mx-cKO mice lacking fibronectin in both hepatocytes and stellate cells to determine which cell type is involved in increased matrix production (shown in figures 2D and 2E). **Experiments in Alb-cKO mice:** (**A**) ELISA for circulating fibronectin shows that deletion of fibronectin in hepatocytes only by using the albumin promoter to drive cre expression (Alb-cKO) results in decreased circulating fibronectin levels in Alb-cKO mice. Fibrosis does not result in an increase in the amount of circulating fibronectin in CT or Alb-cKO compared to healthy mice of the same genotype. The same relationship was true for Mx-cKO mice (shown in Figure 2A). N = 7–8 mice/group. (**B**) Collagen quantification based on hydroxyproline measurements shows that collagen increases in fibrotic animals but unlike Mx-cKO mice (Figure 2E) there is no difference between fibrotic Alb-cKO and fibrotic CT animals in the absence of fibronectin in hepatocytes only. Triplicate measurements/mouse were performed. N = 11–13 mice/fibrotic group and 3 mice/healthy group. **Experiments performed in Mx-cKO mice** lacking fibronectin production in both the hepatocytes and the stellate cells: (**C**) Administration of plasma fibronectin (pFN) at the same time as fibrosis induction does not affect the degree of fibrosis in CT mice or Mx-cKO mice. Mice received daily injections of fibronectin (1 mg/mouse) beginning one day before start of fibrosis induction with DMN. Mice were euthanized after 4 weeks of experimental fibrosis and collagen content in the liver was quantified. N = 4 mice/group. (**D**) The number of inflammatory cells is similar between fibrotic mice lacking fibronectin in hepatocytes and stellate cells (Mx-cKO) and fibrotic controls. Upper panel: Representative pictures showing almost equivalent numbers of inflammatory cells (stained with CD45^+^) in the two fibrotic groups and in the two healthy groups. *Bars represent 100* *µm.* Lower panel: Quantification of the number of CD45^+^ cells. N = 3 mice/group. (**E**) Increased number of α-SMA stained cells and hence activated stellate cells in fibrotic Mx-cKO mice compared to fibrotic controls. Upper panel: Representative pictures showing increased α-SMA stained cells in Mx-cKO mice compared to controls in both the healthy and the fibrotic groups. *Bars represent 100* *µm.* Lower panel: There is a significant increase in the number of α-SMA positive cells in the absence of fibronectin in stellate cells (in Mx-cKO mice). N = 4 mice/group. (**F**) Proliferation of stellate cells in Mx-cKO mice is increased. Co-staining of liver sections with ki-67 (to identify proliferating cells) in red and GFAP (to identify stellate cells) in green was performed. Upper panel: A single cell is enlarged to show how the cells were analyzed. Only cells stained positive for GFAP and showing the characteristic pink color in the overlay of ki-67 and DAPI were counted as proliferating stellate cells. Lower panel: More proliferation was found in fibrotic Mx-cKO mice. N = 3–4 mice/group.

The Mx promoter is able to delete fibronectin in stellate cells, hepatocytes, Kupffer cells and endothelial cells as shown above [15] (Figure 1). In order to exclude a role of fibronectin produced by hepatocytes themselves on the development of fibrosis in Mx-cKO mice we deleted fibronectin in hepatocytes exclusively using the albumin promoter (Alb-cKO). Loss of circulating fibronectin due to successful deletion in hepatocytes was confirmed using ELISA (Figure 3A). The degree of liver fibrosis in Alb-cKO and CT was similar, as determined by measuring the amount of collagen (Figure 3B). Therefore, the absence of fibronectin in hepatocytes in Alb-cKO does not affect the development of fibrosis. We also administered circulating plasma fibronectin to both CT and Mx-cKO mice, and were able to normalize the levels of circulating fibronectin in Mx-cKO mice (Figure S3). This did not affect the degree of fibrosis in CT mice or prevent the increased matrix accumulation in Mx-cKO mice in comparison to fibrotic CT mice (Figure 3C). It follows that lack of fibronectin production in hepatocytes of Mx-cKO mice, and loss of circulating fibronectin do not contribute to enhanced fibrogenesis in these mice.

#### Inflammatory cells

We then sought to determine whether inflammatory cells were involved in mediating increased fibrosis in Mx-cKO mice. An increase in inflammatory cells (CD45^+^) was noted in fibrotic mice compared to healthy mice but there was no difference between Mx-cKO and CT in either the healthy or the fibrotic pairs (Figure 3D). This indicates that fibronectin does not affect the infiltration of inflammatory cells in response to DMN.

#### Hepatic stellate cells

In order to examine the role of stellate cells on fibrosis development in Mx-cKO mice staining for α-SMA was performed. α-SMA has been used as a marker for stellate cell activation but it also stains smooth muscle cells [17]. There was a significant increase in the number of α-SMA-positive cells in both fibrotic and healthy Mx-cKO mice compared to fibrotic and healthy controls respectively (Figure 3E). This increase was associated with an increase in proliferation (as evidenced by co-staining for GFAP and Ki-67) in fibrotic Mx-cKO mice compared to fibrotic CT mice (5% vs. 1%, p<0.05) (Figure 3F). In contrast there was no difference in apoptosis (as evidenced by co-staining for GFAP and TUNEL and for GFAP and cleaved caspase 3 (ASP175)) between fibrotic Mx-cKO mice and fibrotic CT mice (Figure S4 and data not shown). We therefore conclude that the increase in matrix accumulation in Mx-cKO is associated with an increase in proliferation and activation of stellate cells *in vivo*.

### Establishing a causal relationship between deletion of fibronectin in stellate cells and increased matrix production

#### Response of cKO stellate cells to TGF-β

**Figure 4:**
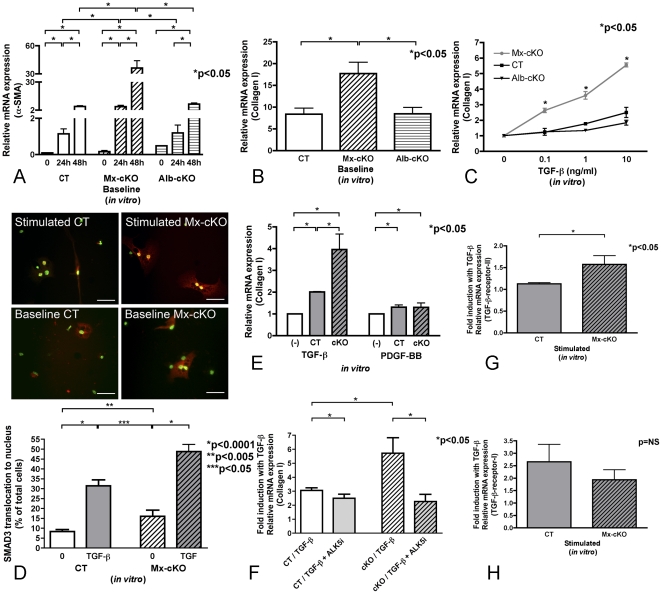
Expression of collagen-type-I mRNA and TGF-β signal transduction in stellate cells in vitro. Hepatic stellate cells were isolated from control mice and Mx-cKO mice in which fibronectin was deleted *in vivo*, and cultured on plastic with media containing fibronectin-depleted FCS. (**A**) Expression of α-SMA increases significantly in Mx-cKO stellate cells compared to CT cells (FN fl/fl) and Alb-cKO cells (Alb-cre FN fl/fl) after 24 hours and increases further after 48 hours in culture (Note the different scales on the three segments of the left y axis). (**B**) Baseline (unstimulated) collagen-type-I mRNA expression is increased in stellate cells isolated from conditional knockout mice (Mx-cKO) when compared to controls (CT and Alb-cKO) as detected by quantitative real time PCR (qPCR) collected from cells after 24 hours in culture. (**C**) Stimulation with up to 10 ng/ml TGF-β for 24 hours results in more pronounced collagen-type-I mRNA expression in Mx-cKO cells. (**D**) SMAD3 translocation is increased in both untreated and stimulated cKO cells. SMAD3 translocation from the cytoplasm to the nucleus was examined in cells left untreated (baseline) for 26 hours after isolation or stimulated with 10 ng/ml TGF-β added 24 hours after culture and left 2 hours on the cells. SMAD3 staining is shown in red and the DAPI-stained nuclei are presented in green. Whenever translocation takes place nuclei are stained yellow due to the overlay of SMAD3 and DAPI. Upper panels: More translocation of SMAD3 takes place in Mx-cKO stellate cells compared to CT at baseline (no TGF-β treatment) and after TGF-β stimulation. *Bars represent 20* *µm.* Lower panel: Graph of the percentage of yellow nuclei in CT (two bars on the left) and cKO cells (on the right) left untreated (white bars) and after stimulation with TGF-β (grey bars). N = 6 wells/group from three experiments. (**E**) PDGF-BB increases collagen-type-I mRNA expression in both Mx-cKO and CT cells, but does not result in increased collagen mRNA expression in cKO compared to CT cells, while TGF-β does. Isolated stellate cells were cultured for 24 hours on plastic then stimulated with either 100 ng/ml PDGF-BB or 10 ng/ml TGF-β for 24 hours. Collagen mRNA expression in untreated cells was normalized to 1 for both TGF-β and PDGF experiments to simplify comparison. N =  at least 5 wells/condition. (**F**) Inhibition of TGF-β signaling (SB431542) results in normalization of the response to TGF-β in cKO cells; there is no significant difference between CT and Mx-cKO cells that were pretreated with the inhibitor and TGF-β (p = NS). Cells were treated for one hour with 10 µM of the inhibitor, followed by 10 ng/ml of TGF-β in the presence of the inhibitor. Collagen-type-I mRNA expression was determined in cells collected 2 hours after the addition of TGF-β. Data shown represent the fold induction in response to either TGF-β or the inhibitor followed by TGF-β after adjustment to baseline (no TGF-β treatment) for ease of comparison. N = 7-9 wells/group. Baseline data are presented in Figure S7. (**G**) TGF-β-receptor-II relative mRNA expression is significantly increased in Mx-cKO stellate cells stimulated with 10 ng/ml of TGF-β for 24 hours compared to CT cells. N = 6 wells/group. (**H**) TGF-β-receptor-I relative mRNA expression is similar between stimulated Mx-cKO and CT stellate cells *in vitro*. N = 6 wells/group.

As shown previously, *in vivo* data had suggested enhanced stellate cell activation and increased collagen production in both healthy and fibrotic Mx-cKO mice compared to their respective controls (Figures 3E and 2E). In order to firmly establish a role for stellate cells lacking fibronectin in mediating increased matrix production *in vivo*, these cells were isolated and examined *in vitro*. α-SMA expression as measured by quantitative real time PCR (qPCR) revealed that Mx-cKO cells had a higher baseline expression of α-SMA after 24 hours of culture on plastic and even more so after 48 hours compared to controls (FN fl/fl or Alb-cre FN fl/fl lacking fibronectin in the circulation only) (Figure 4A), suggesting increased activation. Collagen-type-I relative mRNA expression in cultured stellate cells at baseline was also significantly increased in Mx-cKO cells when compared to CT or Alb-cKO, as measured by qPCR (Figure 4B). We then stimulated stellate cells with TGF-β because it is a major fibrogenic mediator in the liver [2], [18]. Stellate cells cultured on plastic for 24 hours after isolation in the presence of fibronectin-depleted serum were treated with different doses of TGF-β for an additional 24 hours. This resulted in significantly higher collagen-type-I mRNA expression in Mx-cKO compared to both types of control cells (CT and Alb-cKO) at all doses tested (Figure 4C). For example, at 10 ng/ml of TGF-β there was 5-fold induction in Mx-cKO cells compared to 2-fold induction in CT cells in relative mRNA expression of collagen-type-I after correcting for baseline differences (p<0.05, Figure 4C). In contrast, stimulation of hepatocytes with TGF-β failed to show an increase in collagen-type-I expression or a difference between Mx-cKO and CT cells at the three doses tested (data not shown). Lastly, stimulation of either Kupffer cells or endothelial cells failed to show a difference in the response to TGF-β between CT and Mx-cKO cells (Figures S5 and S6). This shows that the absence of fibronectin increases the responsiveness of stellate cells to TGF-β stimulation.

#### Specificity of increased responsiveness of cKO cells

We then sought to determine whether the increase in responsiveness of cKO cells was specific for TGF-β, and therefore performed three sets of experiments. In the first set of experiments, we examined whether the increase in responsiveness to TGF-β is translated into increased intracellular signaling specific for TGF-β. Therefore, translocation of SMAD3 to the nucleus was studied [18], [19]. At baseline the percentage of nuclei in which SMAD3 translocation to the nucleus took place (shown as yellow due to overlay of red SMAD3 and green DAPI staining) was higher in cKO cells (baseline CT: 8% vs. cKO: 16%, p<0.005). Consistently, after stimulation more translocation took place in cKO cells (CT: 32% vs. cKO: 49%, p<0.0001 for CT and cKO compared to the respective baseline and p<0.005 compared to each other) (Figure 4D).

In the second set of experiments we sought to determine whether cytokines other than TGF-β cause increased collagen mRNA expression in cKO cells. Neither treatment with PDGF-BB nor EGF reproduced the effects seen with TGF-β (Figure 4E and data not shown).

In the third set of experiments TGF-β signaling was inhibited in both CT and cKO cells to determine whether a more pronounced inhibition ensued in cKO cells. Adding an inhibitor of TGF-β signaling (SB431542) to cKO cells one hour before treating with TGF-β resulted in a significant decrease in collagen-type-I mRNA expression compared to TGF-β treatment alone in both CT and cKO cells (p<0.05, Figure 4F). Furthermore, the inhibitor resulted in collagen expression in inhibitor-treated cKO cells that was equivalent to inhibitor-treated CT cells (p = NS, Figure 4F). Unstimulated cells showed similar relationships (Figure S7).

We then asked whether this specific response was associated with increased TGF-β-receptor mRNA expression. Indeed, we found that the relative mRNA expression of TGF-β-receptor-II increased in stimulated cKO cells *in vitro* (Figure 4G) while the relative mRNA expression of TGF-β-receptor-I (Alk-5) was not significantly altered between cKO and CT cells *in vitro* (Figure 4H).

In summary, the increase in responsiveness in cKO was specific for TGF-β as was shown in three different experiments.

#### TGF-β amounts in cKO stellate cells

**Figure 5:**
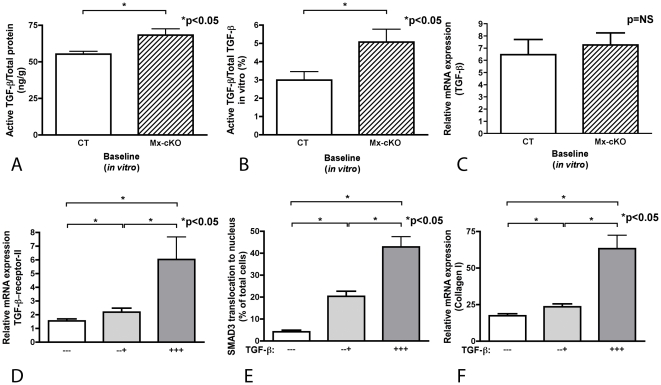
TGF-β content and signaling in vitro. (**A**) Active TGF-β is increased in conditioned media from unstimulated Mx-cKO stellate cells in culture compared to CT cells. After isolation, stellate cells were cultured for 24 hours on plastic, media were then changed and conditioned media (using fibronectin-depleted serum) were collected after an additional 24 hours. Active TGF-β was determined by ELISA in the media. Total protein was determined in cell lysates. N = 7 wells/group. (**B**) The amount of active TGF-β corrected to total TGF-β was increased in Mx-cKO cultures because total TGF-β did not differ in both groups (Data not shown). Active TGF-β was determined in the conditioned media and total TGF-β was determined in the medium together with the cell layer. N = 10 CT and 8 Mx-cKO pairs. (**C**) No difference in relative TGF-β mRNA expression by qPCR between untreated Mx-cKO and CT cells is found. N = 10 wells/group. (**D**) Repeated exposure to 10 ng/ml TGF-β increases TGF-β-receptor-II mRNA expression in wild-type stellate cells in culture compared to one time exposure only. (−−−): Cells cultured for 24 hours (time 0) were left untreated for an additional 24 hours, (−− +): Cells were left untreated at time 0 and 16 hours later, and were stimulated with TGF-β at 20 hours. Cells were stained and mRNA collected after 24 hours, (+ + +): Cells were stimulated with TGF-β at time 0 and then after 16 and 20 hours, and cells were stained and mRNA collected after 24 hours. N = 5 wells/condition. (**E**) Same experimental design was used as in D, and the graph shows that repeated exposure to TGF-β results in increased SMAD3 translocation to the nucleus. N = 4 wells/condition. (**F**) Same experimental design was used as in D, and the graph shows that repeated exposure to TGF-β results in increased collagen-type-I mRNA expression in comparison to one time exposure only. N = 5 wells/condition.

Because of increased responsiveness of cKO cells at baseline we sought to examine whether TGF-β amounts were different between cKO and CT cells. Active TGF-β protein concentrations in conditioned media from hepatic stellate cells cultured for 24 hours on plastic without stimulation were increased in Mx-cKO cells compared to CT as determined by ELISA and confirmed in a bioassay (p<0.05, Figure 5A and data not shown). The amounts of total TGF-β (including matrix TGF-β) were not significantly different between both groups and consequently the percentage of active to total TGF-β was significantly increased in Mx-cKO cells (Figure 5B). This increase could not be directly attributed to an increase in TGF-β mRNA amounts, since TGF-β relative mRNA expression was similar between cKO and CT cells (p = NS, Figure 5C).

#### Establishing a causal relationship between increased active TGF-β levels and increased responsiveness to TGF-β

The finding of an increase in active TGF-β levels does not explain why stellate cells lacking fibronectin exhibit increased sensitivity compared to control cells when stimulated with equivalent concentrations of TGF-β *in vitro* (as shown in Figure 4C). To address this we sought to determine whether TGF-β signaling and collagen-type-I mRNA expression and consequently responsiveness of cKO stellate cells could be affected by increased exposure to TGF-β. In fact, a positive feedback loop of TGF-β on the stellate cells has been proposed [20]. We therefore examined *in vitro* whether repeated exposure of wildtype stellate cells to TGF-β leads to increased TGF-β-receptor-II mRNA expression and SMAD3 translocation to the nucleus, which then in turn will lead to increased collagen-type-I expression. Increased TGF-β-receptor-II mRNA expression and SMAD3 translocation were more pronounced after three exposures to TGF-β (-24, -8, -4 hours) than after a single exposure (-4 hours) (p<0.05, Figures 5D and 5E). This was associated with an increase in collagen-type-I mRNA expression (p<0.05, Figure 5F). These findings thus suggest that an increase in available active TGF-β is associated with increased responsiveness of stellate cells to TGF-β.

### Changes in TGF-β protein amounts and signaling in cKO mice

**Figure 6:**
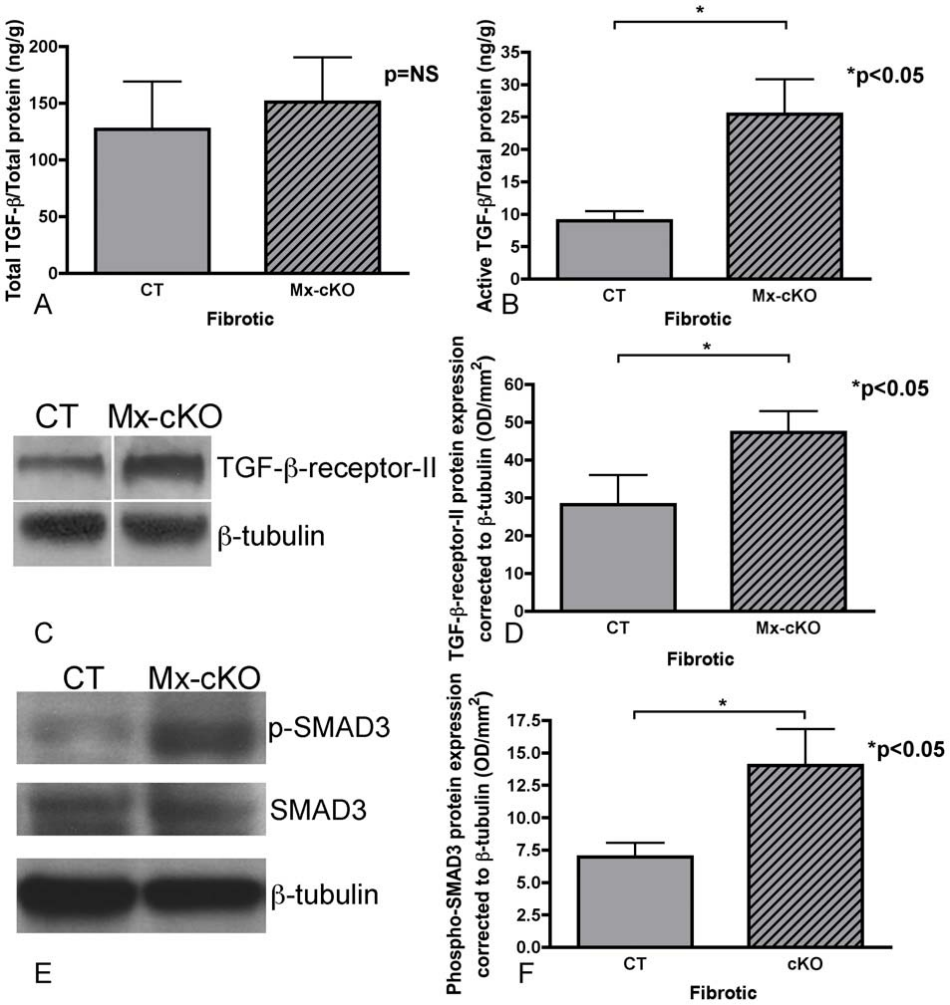
TGF-β content and signaling in vivo after induction of liver fibrosis in CT and Mx-cKO mice. (**A**) Total TGF-β in liver lysates was similar between CT and Mx-cKO mice. TGF-β was determined using a commercial assay, and corrected to total protein determined by BCA as described in the methods. N = 6/group. (**B**) Active TGF-β was increased in Mx-cKO livers in comparison to CT. Active TGF-β was determined using liver lysates from the same mice as above and corrected to total protein. (**C**) TGF-β-receptor-II protein expression is increased in liver tissue from fibrotic Mx-cKO mice compared to fibrotic CT mice. A Western blot is shown for a representative pair with β-tubulin used as a loading control. (**D**) Densitometry for protein expression of TGF-β-receptor-II in liver tissue from fibrotic Mx-cKO and CT mice corrected to β-tubulin. N = 6/group. (**E**) Protein expression of phosphorylated SMAD3 is increased in fibrotic Mx-cKO livers compared to CT *in vivo*. Shown is a representative pair suggesting increased phospho-SMAD3 in cKO *in vivo* (from top: phospho-SMAD3, total SMAD3, and β-tubulin). (**F**) Densitometry for phospho-SMAD3 in Western blotting shows an increase in phospho-SMAD3 when corrected to β-tubulin. N = 6/group.

Based on the described *in vitro* experiments, increased collagen production by the cKO stellate cells was due to an increase in active TGF-β and hence increased responsiveness of these cells. In order to determine whether an increase in available TGF-β was the culprit for boosted fibrosis in Mx-cKO mice, total TGF-β content in fibrotic-Mx-cKO and fibrotic-CT livers was determined by treating mechanically disrupted liver lysates with hydrochloric acid (HCl) to release all TGF-β including matrix-bound TGF-β. However, no difference could be detected (p = NS, Figure 6A). In contrast, active TGF-β in liver lysates was increased in the livers of fibrotic Mx-cKO mice as measured by ELISA (p<0.05, Figure 6B). Because TGF-β that is bound to the latency-associated peptide and hence is inactive, can be detected by antibodies used in ELISA tests, we performed a bioassay that detects only free unbound TGF-β [21]. Similar relationships were obtained (data not shown). The increase in the amount of active TGF-β was associated with enhanced TGF-β-receptor-II expression by Western blotting in livers from fibrotic Mx-cKO mice (Figures 6C and 6D) but no change in TGF-β-receptor-I expression *in vivo* (Figure S8). TGF-β signaling was also augmented as evidenced by increased SMAD3 phosphorylation in fibrotic Mx-cKO livers *in vivo* shown by Western blotting (Figures 6E and 6F). Presumably this ultimately led to boosted collagen production shown in Figure 2E. Hence, these *in vivo* findings replicate the findings in stellate cells *in vitro* and implicate the increase in active TGF-β as the culprit for enhanced collagen production in fibrotic Mx-cKO mice.

### The role of the presence of fibronectin around the cells

**Figure 7:**
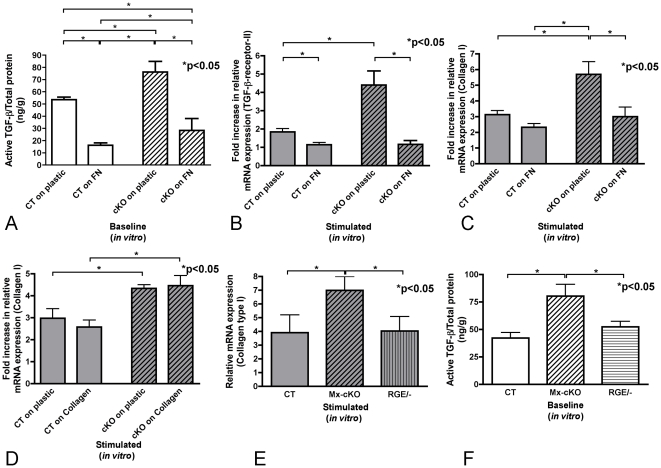
Fibronectin normalizes the responsiveness of cKO cells. (**A**) Active TGF-β in the medium decreases significantly both in control and Mx-cKO cells in the presence of fibronectin coating. Stellate cells were cultured directly after isolation for 24 hours on plates left uncoated or coated with fibronectin, media were changed and collected after an additional 24 hours in culture (using 10% fibronectin-depleted FCS). N = 5 wells/group. (**B**) Fibronectin coating is associated with a decrease in TGF-β-receptor-II mRNA expression in response to TGF-β stimulation in Mx-cKO cells. Isolated cells were cultured on plastic or on fibronectin for 24 hours, then left untreated or treated with 10 ng/ml TGF-β for an additional 24 hours after which mRNA was collected. Data presented are adjusted to unstimulated cells to facilitate comparison. N = 6/group. (**C**) Fibronectin coating normalizes the response of cKO cells to TGF-β stimulation. No difference in the responsiveness to TGF-β stimulation in the presence of fibronectin coating between CT and Mx-cKO cells is seen, while cKO cells cultured on plastic show a higher responsiveness to TGF-β than CT cells on plastic. Cells were cultured and data are presented as in B. N = 6/group. (**D**) Collagen coating does not affect the response of cells to TGF-β. Therefore, cKO cells continue to show an increase in responsiveness to TGF-β that is unrelated to the presence or absence of collagen coating. Cells were cultured and data are presented as in B. N = 4/group. (**E**) The presence of fibronectin fibrils with mutated RGD domain and hence unable to activate integrins via RGD is enough to result in normal amounts of collagen production in response to TGF-β stimulation. Deletion of fibronectin in mice with the genotype Mx-cre/- FN-RGE/fl resulted in cells that only express one mutated fibronectin (RGE/-). Cells were treated with TGF-β and collagen-type-I examined by qPCR. N = 6–7/group. (**F**) The presence of fibronectin with an RGE mutation is enough to normalize the levels of active TGF-β in the conditioned media. Cells were cultured as in A. N = 6–7/group.

The previous data suggest that the absence of fibronectin in the matrix surrounding the stellate cells leads to increased active TGF-β and increased responsiveness of cKO stellate cells. Thus one would expect that the presence of fibronectin in the surrounding of the stellate cells would have two consequences: first, it will result in a decrease in active TGF-β in the media and second, it should protect the cKO cells from excessive stimulation by TGF-β. Wells were therefore pre-coated with fibronectin. The amount of active TGF-β in the conditioned media of stellate cells cultured for 24 hours on fibronectin decreased significantly both in control cells (p<0.05), and in cKO cells (p<0.05, Figure 7A). Thus coating with fibronectin reduced the amount of active TGF-β in the media of cKO cells, but did not completely normalize it. Nevertheless, the decrease was significant compared to controls cultured on plastic (p<0.05). The second consequence, namely that fibronectin coating should lead to normalization of the responsiveness of cKO cells was also confirmed. The TGF-β-induced increase in mRNA expression of TGF-β-receptor-II and collagen type I was blunted in cKO cells cultured on fibronectin (uncoated vs. coated Mx-cKO: p<0.05, coated cKO vs. coated CT: p = NS, coated cKO vs. CT on plastic: p = NS, Figures 7B and 7C). These data thus show a complete restoration of normal responsiveness of cKO cells in the presence of fibronectin in the cell surrounding. This effect was specific for fibronectin, because coating with collagen failed to affect the amount of collagen-type-I mRNA expression in CT or cKO cells after stimulation (Figure 7D).

In order to determine whether the formation of fibrils was enough to normalize the response of stellate cells to TGF-β we used stellate cells from mice that express only one fibronectin gene with a mutated RGD binding domain [22]. These cells were obtained from mice with the following genotype: Mx-cre/- FN-RGE/fl. Activating Mx-cre results in deletion of the floxed fibronectin allele, and the stellate cells can only produce fibronectin that is able to form fibrils but is unable to activate integrins via RGD (RGE/-). Treatment of these cells with TGF-β failed to induce an increased response, as measured by collagen-type-I mRNA expression compared to CT (Figure 7E). This suggests normalization of the response of stellate cells to TGF-β. Indeed, the concentration of active TGF-β in the conditioned media from untreated stellate cells was similar between the cells that produce normal fibronectin (CT) and those that produce the mutated fibronectin (RGE/-) (Figure 7F). This indicates that binding of fibronectin to integrins through the RGD domain is not required for normalization of the stellate cells response to TGF-β, while providing fibronectin fibrils is enough for normalization of the response.

Based on these findings we conclude, that the presence of fibronectin fibrils in the matrix surrounding stellate cells significantly diminishes the amount of active TGF-β released and restores a normal response to TGF-β.

## Discussion

The principal findings of this study are: 1) Deletion of fibronectin in the liver results in increased collagen at baseline and in response to induction of experimental fibrosis. 2) Deletion of fibronectin in hepatic stellate cells leads to increased collagen-type-I at baseline and after activation. 3) These effects are associated with increased active TGF-β protein and cellular response to TGF-β, are mediated by the SMAD pathway, are not due to increased TGF-β mRNA expression, but are associated with increased TGF-β-receptor-II mRNA expression. 4) Inhibiting TGF-β signaling or providing fibronectin to stellate cells *in vitro* normalizes the heightened responsiveness of cKO stellate cells. The increase in fibrosis in mice lacking fibronectin in the liver is thus due to an increase in active TGF-β, most likely resulting from a failure to incorporate TGF-β in the matrix in the absence of fibronectin. These findings show that *in vitro* fibronectin production by hepatic stellate cells is critical for modulating the amount of available bioactive TGF-β and hence the cell responsiveness to TGF-β. This offers a possible explanation for the *in vivo* finding that the presence of fibronectin seems to protect against exacerbation of fibrosis.

Fibronectin is required *in vitro* for the assembly of a collagen matrix [5], [6]. In its absence a reduction in collagen matrix assembly would be expected. This, however, was not the case in our model. Immunohistochemistry of liver sections (data not shown) and ELISA data (Figure 2C) show a residual amount of fibronectin in the matrix, despite the deletion of fibronectin in the circulation and stellate cells by >95%. Presumably, this residual amount originates from the time before induced deletion of fibronectin at 4 weeks postnatally, and might be sufficient to support collagen assembly. It is also possible, that despite the apparent successful deletion of fibronectin the remaining undeleted circulating fibronectin is enough to support normal collagen matrix assembly even though it is much diminished, since circulating fibronectin contributes to tissue fibronectin [7], [23]. A related issue is whether the decrease in the amount of surrounding fibronectin may modulate the synthesis of collagen. Our *in vitro* data show that this is the case since cKO stellate cells unable to produce fibronectin showed a decrease in collagen expression when cultured on fibronectin-coated plates compared to cells cultured on plastic (Figure 7C). Unfortunately, the degree of deletion of circulating fibronectin using the albumin promoter at 91% was less pronounced than using the Mx promoter at 97% in relation to control mice. Theoretically this residual circulating fibronectin might have been enough and hence could have resulted in equivalent degrees of fibrosis in hepatocyte specific conditional knockouts and their controls, but this is unlikely because we could not detect a dose response effect of circulating fibronectin on fibrosis, and normalizing circulating levels of fibronectin failed to prevent the enhanced fibrosis in Mx-cKO mice compared to fibrotic CT mice. Another cell type that is affected by the deletion of fibronectin using the Mx-promoter is the endothelial cell. Sinusoidal endothelial cells exert a profibrotic effect particularly in the early stages of liver fibrosis [24]. It has been shown that early in fibrosis these cells produce a fibronectin isoform called EDA fibronectin that can be detected in sinusoids *in vivo* as well as *in vitro* in endothelial cell cultures. Furthermore, endothelial cells in culture form a fibronectin matrix that accelerates hepatic stellate cell activation [24]. Based on these findings one would have expected a decrease in stellate cell activation in the absence of fibronectin in endothelial cells, and not an increase as presented here. On the other hand, it is conceivable that the absence of fibronectin production by the endothelial cells using the Mx-promoter contributed to the increase in bioavailable TGF-β. At any rate our data clearly implicate the stellate cells both *in vivo* and *in vitro* as a culprit for increased collagen production in our model.

The activated stellate cells produce fibronectin isoforms that differ in their characteristics from plasma fibronectin [3]. While plasma fibronectin represents the soluble form of fibronectin, the three isoforms produced by the stellate cells belong to the group of cellular isoforms that are also viewed as being insoluble, since they are found in high concentrations in the tissue. These three isoforms are: EDA, EDB and oncofetal fibronectin (oFN) (defined by an O-glycosylation site in the variable region) [3], [25], [26]. Only little of these isoforms escapes from the matrix and can be detected in the circulation [27], while plasma fibronectin has no demonstrable role in the formation of fibrosis in any reported model. Under certain circumstances plasma fibronectin is involved in extracellular matrix integrity [25], [28], and a number of *in vitro* studies examining the role of fibronectin in collagen matrix assembly have used plasma fibronectin despite the presence of one report suggesting that the absence of extra-domain-B (EDB) results in fibronectin fibrils that are thinner and shorter [29]. Differences, however, have been reported for the effects on the cells. The EDB domain has been shown to support the proliferation of fibroblasts [29]. The presence of extra-domain-A (EDA) has been implicated in upregulating binding affinity to α5β1-integrin and integrin-mediated signal transduction [8], [30]. It has also been identified as a binding domain for α4β1-integrin [31]. Even though the possibility that an isoform of fibronectin, other than plasma fibronectin may normally inhibit stellate cells cannot be excluded, we are not aware of data to support such a conclusion. If anything there are reports that the presence of fibronectin containing the EDA domain resulted in activation, instead of inhibition of hepatic stellate cells *in vitro* [24], [32]. Together these data support an indirect effect of fibronectin with regards to TGF-β regulation.

An increase in active TGF-β in the absence of an adequate fibronectin matrix can explain all our findings starting with the increase in baseline production of collagen-tsype-I in healthy as well as in fibrotic Mx-cKO mice, the increase in active stellate cell numbers, and finally the increase in TGF-β signaling [2], [33]. At first glance it may seem surprising that fibronectin is able to blunt the response to TGF-β. It has been unequivocally shown however, that fibronectin plays two roles in TGF-β modification. On the one hand, it is required for the incorporation of TGF-β bound to the latent TGF-β binding protein-1 (LTBP-1) in the matrix [34], and on the other hand, it is required for the αvβ6-integrin mediated activation of TGF-β stored in the matrix [35]. Our *in vitro* data show an increase at baseline in bioactive TGF-β without an associated increase in TGF-β mRNA in cKO stellate cells. Thus the data suggest that the incorporation of TGF-β in the matrix, and not the production of TGF-β is abnormal resulting in an increase in the amount of available TGF-β. The fact that total TGF-β was similar between CT and cKO fibrotic livers suggests that in the absence of fibronectin in stellate cells total TGF-β is irrelevant to the pathophysiology of fibrosis. It also appears that the total amount of TGF-β is not modulated by the amount of free TGF-β since in the CT livers 7% of the total TGF-β consisted of active TGF-β, while in cKO livers the percentage was 17%.

Recently accepted but not yet published work by Moriya *et al.* [36] used a similar model and focused on matrix assembly. While we found that there was significant induction of collagen production in Mx-cKO mice at baseline and after DMN and CCl_4_ administration (Figure 2E and data not shown), Moriya et al. found no difference either at baseline or after CCl_4_ treatment. Since our finding was reproducible and could be confirmed using two different models of fibrosis induction we currently lack an explanation for this discrepancy. Both works complement each other in that Moriya *et al.* imply that TGF-β stimulates matrix assembly, while we established both *in vivo* and *in vitro* that the amount of active TGF-β originating from the stellate cells is at least partially responsible for increased matrix production in a fibronectin-deficient state.

In summary, our data support the conclusion that in healthy and injured liver, stellate cells require adequate amounts of matrix fibronectin in order to buffer the amount of bioavailable TGF-β and its fibrogenic outcome.

## Materials and Methods

### Mice

Mice possessing an inducible Mx promoter or an albumin promoter driving cre-recombinase expression were crossed with mice carrying *loxP*-flanked (floxed) fibronectin (FN-fl/fl) or mice carrying a fibronectin gene with a mutated RGD to RGE sequence in the integrin binding domain and the floxed gene on the second allele (FN-RGE/fl) [15], [22], [37]. Mx was induced at 4 weeks of age using 250 µg polyinosinic-polycytidylic acid (pIpC) injected three times over one week as described [15]. Animals used were of the C57Bl/6 background. Fibronectin in plasma or liver lysates was determined by ELISA [25].

### Induction of liver fibrosis

Liver fibrosis was induced by injecting dimethylnitrosamine (DMN) intraperitoneally at 10 µg DMN/g body weight (Sigma-Aldrich) 3x/week for 4 weeks in male mice [13]. Mice in the Mx group started induction of liver fibrosis at the age of 8-9 weeks while mice in the Alb group started induction at the age of 9–11 weeks. Human plasma fibronectin isolated from outdated plasma as described was administered i.p. at a dose of 1 mg/mouse/d [25], [38]. The studies in mice were approved by the animal protection committee of the University of Heidelberg #ma34050, #ma35590, #ma36955, and #A-18/09. Thus, all animal work was conducted according to relevant national and international guidelines.

### Determination of the degree of fibrosis

Morphometric analysis of areas stained with sirius-red was performed using the program ImageJ (Wayne Rasband, NIH). Hydroxyproline was measured using a biochemical method as published [39], [40]. To calculate the amount of collagen, we multiplied measured hydroxyproline content by 10 as proposed by Berg in 1982 [39]. Liver enzymes alanine aminotransferase (ALT) and aspartate aminotransferase (AST) were measured in serum using routine clinical chemistry.

### Cell culture

Hepatic stellate cells, Kupffer cells and endothelial cells were isolated from Mx-conditional knockout mice (Mx-cKO) which carry the Mx-promoter attached to cre and are homozygous for the floxed fibronectin gene [15], [37]. For some experiments mice that carry Mx-cre, a mutated fibronectin gene that contains an RGE domain instead of the RGD domain and a floxed fibronectin gene were used (Genotype: Mx-cre/- FN-RGE/fl) [22]. In all Mx mice the Mx promoter was activated *in vivo* prior to cell isolation as described [10]. Cells from littermates lacking the Mx-cre gene or carrying the Alb-cre gene together with two floxed fibronectin genes were used as controls. Briefly, the liver was perfused and the cells were separated using Nycodenz (Axis-shield) as described [41]. Cells were cultured in DMEM and 10% fibronectin-depleted fetal-calf-serum (FCS) [10]. Depletion of FCS was performed by running FCS on gelatine-sepharose columns 3-5 times. Final concentration of fibronectin was 8-11 ng/ml in the batches used. All experiments were started 24 hours after isolation. Unless noted otherwise, cells were treated with TGF-β_1_ (1-10 ng/ml), murine platelet derived growth factor-BB (PDGF-BB) (100 ng/ml) (Sigma-Adrich) or murine epidermal growth factor (EGF) (100 ng/ml) (Peprotech). The TGF-β signaling inhibitor (SB431542) (Merck) was used at a final concentration of 10 µM [42]. Treatment with TGF-β_1_, PDGF-BB or EGF lasted for 24 hours or as described in the figure legends, and treatment with the inhibitor lasted one hour, after which TGF-β was added and left for an additional 2 hours before collecting the samples. Fibronectin used for coating was isolated from outdated human plasma as described [25], and collagen was from human placenta (Sigma-Aldrich). Both were added at 100 µg/ml overnight, because it has been reported that high concentrations of collagen are associated with stimulation of stellate cells [43].

### DNA analysis

DNA was isolated from stellate cells using proteinase K and nested PCR was performed using the following primer pairs consecutively: fibronectin forward-5′-TCGCACCCGCTGCGCTGCA-3′, reverse-5′-ATTGTCAAAACAGCCAGCTGCGA-3′, and then forward-5′-AGGGTGTGAGCCGGACAACT-3′, reverse-5′-CAACTGACTTGGTGAGCCTGCA-3′.

### RNA analysis

RNA was isolated using RNeasy (Qiagen). Quality was tested using RNA-6000-Pico-Chip (Agilent). Reverse transcription was performed using iScript-Select (BioRad). The following primer pairs were used: fibronectin forward-5̀-GTCAGTGTCTCCAGTGTCTAC-3′, reverse-5′-TGGCTTGCTGGCCAATCAGT-3′, desmin forward-5′-CTGGAGGCTTGGGGTCGC-3′, reverse-5′-CGGATCTCCTCTTCATGCAC-3′, glial fibrillary acidic protein (GFAP) forward-5′-AGCGAGCGTGCAGAGATGAT-3′, reverse-5′- CCGAGGTCCTGTGCAAAGT-3′, GAPDH forward-5′-TGAAGGTCGGTGTGAACGGATTT-3′, reverse-5′-CATGTAGGCCATGAGGTCCAC-3′.

Quantitative real time PCR (qPCR) products were obtained using LightCycler and TaqMan probes, and results were normalized to hypoxanthine-phosphoribosyltransferase (HPRT) after standard curves were established. The choice of HPRT for normalization was based on early experiments showing comparable expression between CT and Mx-cKO liver. Data were evaluated using RelQuant (Roche). The primers used were those suggested by the universal primer library from Roche. The probes were as follows: Collagen-type-I and TGF-β_1_ probe #15, HPRT probe #95, TGF-β-receptor-I probe#32, TGF-β-receptor-II probe#7, α-SMA probe #21, C-terminal fibronectin probe #66, EDA fibronectin probe #76, and EDB fibronectin probe #31.

### TGF-β ELISA and bioassay

Mouse TGF-β1 was determined using an ELISA kit from Promega and confirmed using a bioassay. This was done because the ELISA antibodies may detect TGF-β that is bound to the latency-associated peptide (LAP), and hence inactive. In the bioassay mink lung epithelial cells (MLEC) stably transfected with an expression construct containing a truncated PAI-1 promoter fused to the firefly luciferase reporter gene are responsive only to free active TGF-β, and hence this assay by virtue of its design only detects bioavailable TGF-β.

Conditioned media from stellate cells in culture for 24 hours were used to determine active TGF-β. This was diluted 1∶5 for the commercial assay and 1∶4 for the bioassay. In order to determine total TGF-β *in vitro* stellate cell plates were heated to 80°C for 10 minutes and then placed on ice. Supernatants including the lysed cells were then assayed (using the kit) or added to the reporter cells in a dilution of 1∶4 (100 µl) for 5 hours and luciferase assayed as described [21]. Bioluminescence was measured in white plates using Wallac-Victor2-1420 (Perkin-Elmer). The amount of active or total TGF-β was then corrected to total protein measured using the BCA kit (Pierce).

In order to determine TGF-β in liver tissue using the commercial assay, liver pieces (50-100 mg in 300 µl DPBS) were mechanically disrupted using a Hybaid-Ribolyser-cell-disrupter, half was then treated with HCl followed by neutralization with NaOH as suggested in the manual to determine total TGF-β. The other half was used without HCl treatment to determine active TGF-β and total protein. Both treated and untreated samples were tested at a final dilution of 1∶10. For the bioassay, liver pieces were disrupted in 500 µl DMEM, half was centrifuged at 13000 RPM for 15 minutes and supernatant used for measuring active TGF-β and total protein, and the second half was heated to 80°C for 10 minutes followed by 10 minutes on ice and 1 minute centrifugation. The supernatant was then used for total TGF-β and total protein determinations. Liver lysates were diluted 1∶400. Bioluminescence was measured as described above and corrected to total protein.

### Staining protocols and immunohistochemistry

Extracellular matrix was stained using picro-sirius-red in 5 µm frozen sections of the liver as reported [44]. Cells or liver sections were fixed in 4% paraformaldehyde or ice-cold acetone. The following antibodies were used: rabbit anti-GFAP-IgG (Acris); mouse anti-desmin-IgG (AbD Serotec); rat anti-CD45-IgG and mouse anti-ki-67-IgG conjugated with alexa-555 (BD Pharmingen); rat anti-F4/80 (E-Bioscience); rabbit anti-cleaved caspase 3 (ASP175) conjugated with alexa-488 (Cell Signaling); mouse anti-α-SMA-IgG conjugated with Cy3 (Sigma-Aldrich); rabbit anti-SMAD3-IgG (Cell Signaling); rabbit anti-mouse fibronectin (Millipore); rabbit anti collagen-type-I (Abcam); goat anti-rabbit conjugated with Cy2; goat anti-rabbit conjugated with alexa-555 (Molecular Probes); goat anti-mouse-IgG conjugated with Cy2 (Dianova), and goat anti-rat-IgG conjugated with Cy2 (Dianova). Slides were photographed in the Nikon Imaging Center (University of Heidelberg) and processed using ImageJ (Wayne Rasband, NIH). TUNEL was performed according to the manufacturer's recommendations (Trevigen). Endothelial cells were stained by adding Dil-AcLDL (Invitrogen) at a concentration of 10 µg/ml to living cells for 4 hours at 37°C. Quantification was performed in at least three sections per mouse in at least three mice per group or more as noted in the figure legends. At least 200 positive cells were counted per section and then corrected to the area examined.

### Western blotting and immune precipitation

Fibronectin and SMAD3 were detected using the same antibodies as above. In addition, the following antibodies were used: anti-TGF-β-receptor-II (Abcam); anti-TGF-β-receptor-I (R-20) (Santa Cruz); rabbit anti-phospho-SMAD3-IgG (Cell Signaling), and anti-β-Tubulin (Sigma-Aldrich). Western blotting of fibronectin in stellate cells *in vitro* was preceded by immune precipitation, which was performed using gelatine-sepharose beads added to equivalent protein amounts of cell lysates from cKO and CT cells for 1 hour. After washing, Laemmli buffer was added to the coated beads, which were boiled before being applied to the gel. The beads did not require coating with an antibody because of the avid binding of fibronectin to them. All antibodies were diluted in 5% BSA, except fibronectin which was diluted in 5% milk powder/tris-buffered saline.

### Statistical analyses

Analyses were performed using SPSS (V14.0) and first applying ANOVA tests when appropriate. Comparisons were then performed using Student's *t*-test or Wilcoxon paired test as appropriate. Results are expressed as mean±standard-error-of-the-mean (M±SEM).

## Supporting Information

Figure S1 **Expression of C-terminal fibronectin and the EDA and EDB domains in fibrotic livers of CT and Mx-cKO mice.** Relative mRNA expression in the liver of fibrotic CT and Mx-cKO mice was determined by quantitative real time PCR for C-terminal fibronectin, EDA, and EDB domains. The percent values over Mx-cKO bars show the relative remaining expression of mRNA for the three products.
(TIF)

Figure S2 **Effect of fibrosis on the circulating levels of AST and ALT.** Despite the more pronounced increase in collagen in fibrotic Mx-cKO mice there is no difference in liver enzymes AST or ALT between fibrotic Mx-cKO mice and fibrotic CT mice reflecting absence of a more pronounced increase in necrosis of hepatocytes in fibrotic Mx-cKO mice.
(TIF)

Figure S3 **Normalization of circulating levels of fibronectin in Mx-cKO mice, in which fibrosis was induced and which received daily injections of fibronectin at 1 mg/mouse/day in comparison to control mice (CT) in which fibrosis was induced, but which did not receive daily fibronectin injections.** Blood was drawn and tested 10 days after the start of injection. N = 4/group.
(TIF)

Figure S4 **Apoptosis of stellate cells is similar between fibrotic CT and fibrotic Mx-cKO mice lacking fibronectin.** Liver sections were co-stained with TUNEL (to detect apoptotic cells) and GFAP (to detect stellate cells). Quantification of the percent apoptotic stellate cells revealed no difference between the two fibrotic groups or between the two healthy groups. At least 1800 cells were counted per mouse in the fibrotic groups, and 800 cells in the healthy groups. 3 mice were analyzed per group.
(TIF)

Figure S5 **Deletion of fibronectin in Kupffer cells does not result in increased responsiveness to TGF-β.** Cells were left untreated or treated on the same day of isolation with 10 ng/ml of TGF-β in medium with 10% fibronectin-depleted FCS. Twenty four hours later RNA was collected and qPCR was performed. Results are expressed as fold increase over baseline. N = 6/group.
(TIF)

Figure S6 **Deletion of fibronectin in endothelial cells does not result in increased responsiveness to TGF-β.** Isolated endothelial cells were left untreated or treated on the day following isolation with 10 ng/ml of TGF-β in medium with 10% fibronectin-depleted FCS. Twenty four hours later RNA was collected and qPCR was performed. Results are expressed as fold increase over baseline. N = 6/group.
(TIF)

Figure S7 **Effect of inhibiting TGF-β-receptor-I on baseline collagen-type-I mRNA expression.** Adding an inhibitor of TGF-β-receptor-I (SB-431542) to cKO cells resulted in a significant decrease in baseline collagen-type-I mRNA production by 50% (p<0.05) compared to untreated cKO cells, while collagen mRNA expression failed to show a significant difference between untreated CT cells and those treated with the inhibitor only. Furthermore, no significant difference in baseline collagen mRNA expression is found between CT cells treated with the inhibitor only and cKO cell treated with the inhibitor only. Cells were cultured for 24 hours on plastic after isolation, either left untreated or treated with 10 µM of the inhibitor and collected 3 hours later. N = 8 wells/group for CT cells and 6 wells/group for cKO cells.
(TIF)

Figure S8 **TGF-β-receptor-I protein expression in liver tissue from fibrotic Mx-cKO mice is not increased compared to liver tissue from fibrotic CT mice.** **(A)** shows a representative pair from a Western blot for TGF-β-receptor-I. β-tubulin was used as a loading control. **(B)** shows densitometry of protein expression from 6 CT and 6 Mx-cKO livers.
(TIF)
