# Supplementary figures and images for: Fibronectin Protects from Excessive Liver Fibrosis by Modulating the Availability of and Responsiveness of Stellate Cells to Active TGF-β
Source: PLoS One. 2011 Nov 28;6(11):e28181. doi: 10.1371/journal.pone.0028181 (PMC3225392; doi:10.1371/journal.pone.0028181)

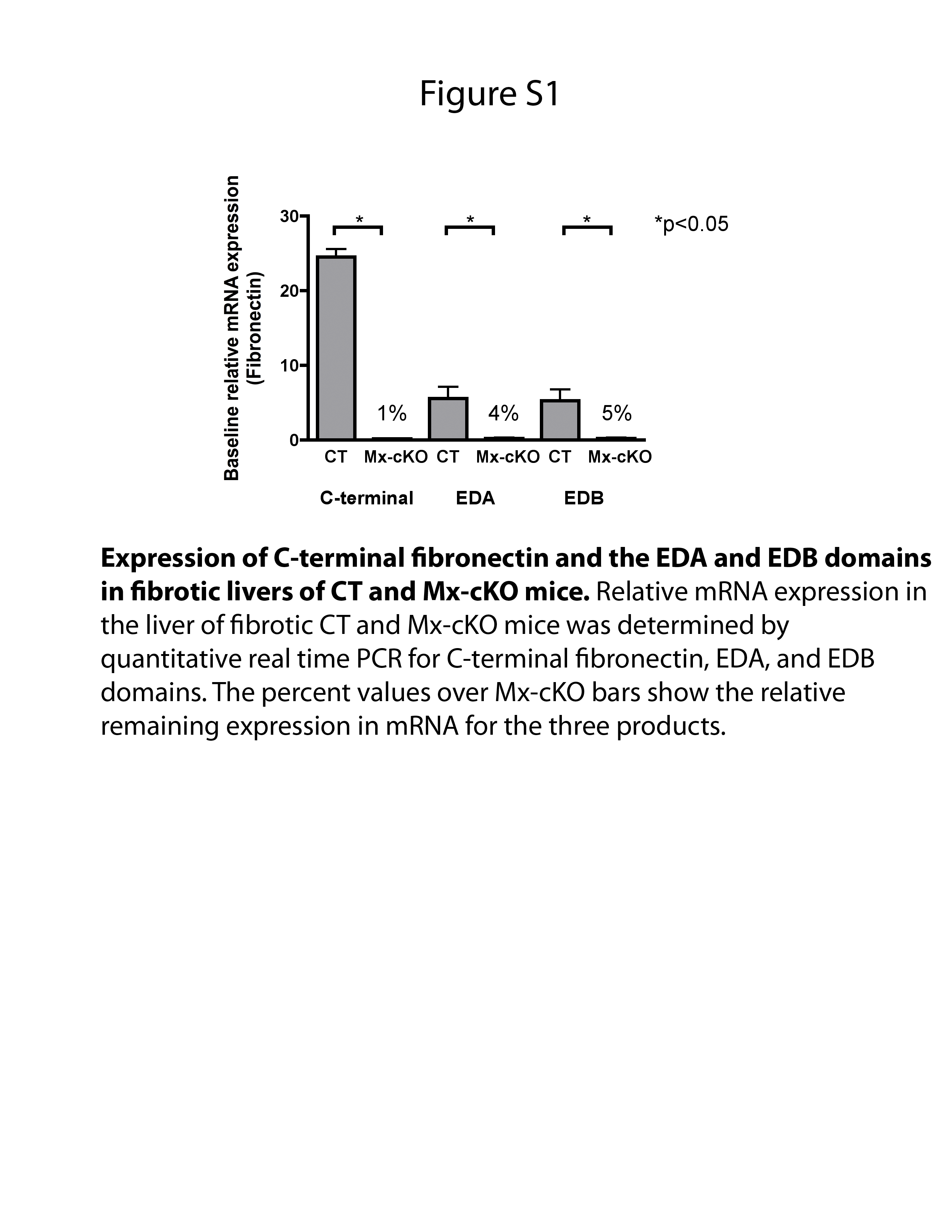

Supplement: Figure S1 — Expression of C-terminal fibronectin and the EDA and EDB domains in fibrotic livers of CT and Mx-cKO mice. Relative mRNA expression in the liver of fibrotic CT and Mx-cKO mice was determined by quantitative real time PCR for C-terminal fibronectin, EDA, and EDB domains. The percent values over Mx-cKO bars show the relative remaining expression of mRNA for the three products. (TIF) [file pone.0028181.s001.tif]

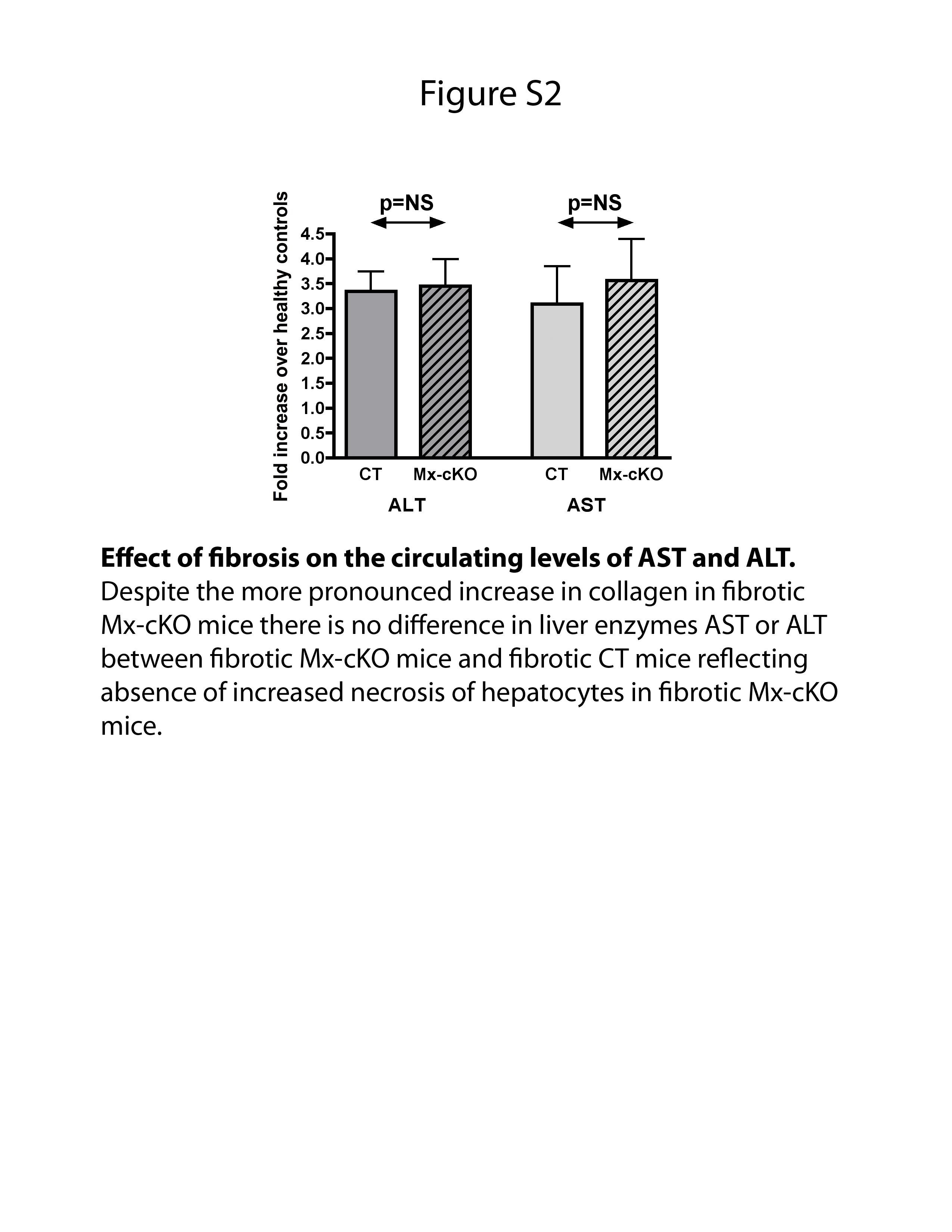

Supplement: Figure S2 — Effect of fibrosis on the circulating levels of AST and ALT. Despite the more pronounced increase in collagen in fibrotic Mx-cKO mice there is no difference in liver enzymes AST or ALT between fibrotic Mx-cKO mice and fibrotic CT mice reflecting absence of a more pronounced increase in necrosis of hepatocytes in fibrotic Mx-cKO mice. (TIF) [file pone.0028181.s002.tif]

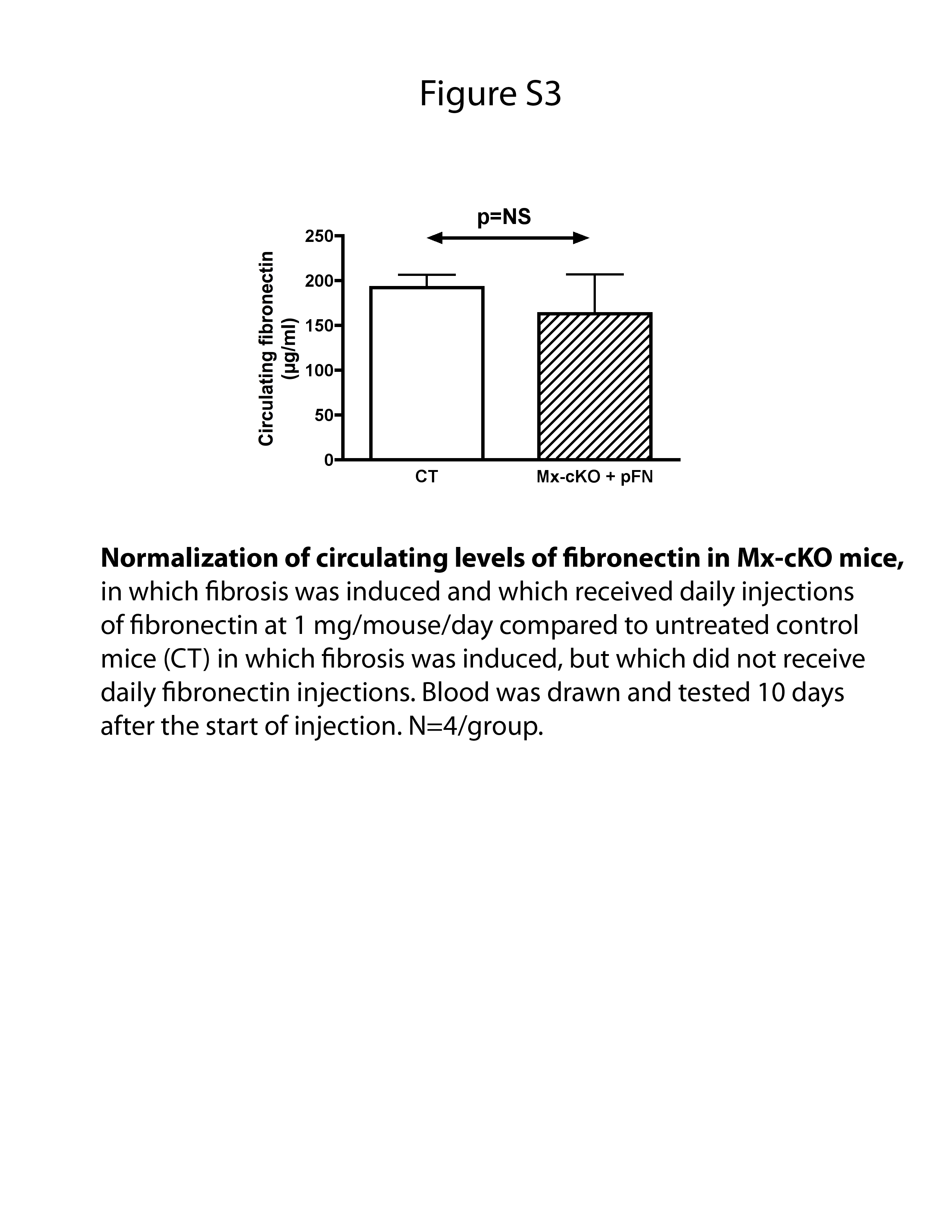

Supplement: Figure S3 — Normalization of circulating levels of fibronectin in Mx-cKO mice, in which fibrosis was induced and which received daily injections of fibronectin at 1 mg/mouse/day in comparison to control mice (CT) in which fibrosis was induced, but which did not receive daily fibronectin injections. Blood was drawn and tested 10 days after the start of injection. N = 4/group. (TIF) [file pone.0028181.s003.tif]

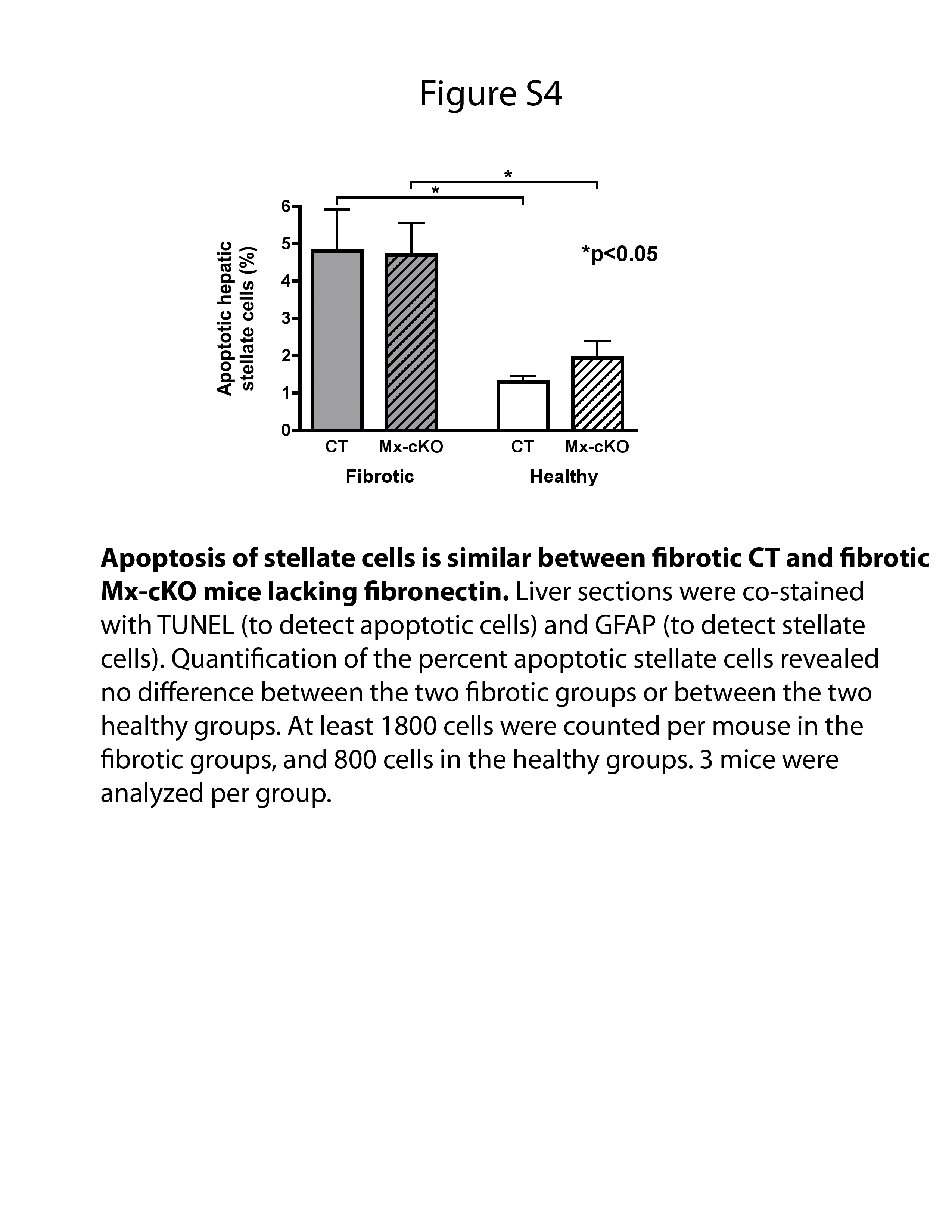

Supplement: Figure S4 — Apoptosis of stellate cells is similar between fibrotic CT and fibrotic Mx-cKO mice lacking fibronectin. Liver sections were co-stained with TUNEL (to detect apoptotic cells) and GFAP (to detect stellate cells). Quantification of the percent apoptotic stellate cells revealed no difference between the two fibrotic groups or between the two healthy groups. At least 1800 cells were counted per mouse in the fibrotic groups, and 800 cells in the healthy groups. 3 mice were analyzed per group. (TIF) [file pone.0028181.s004.tif]

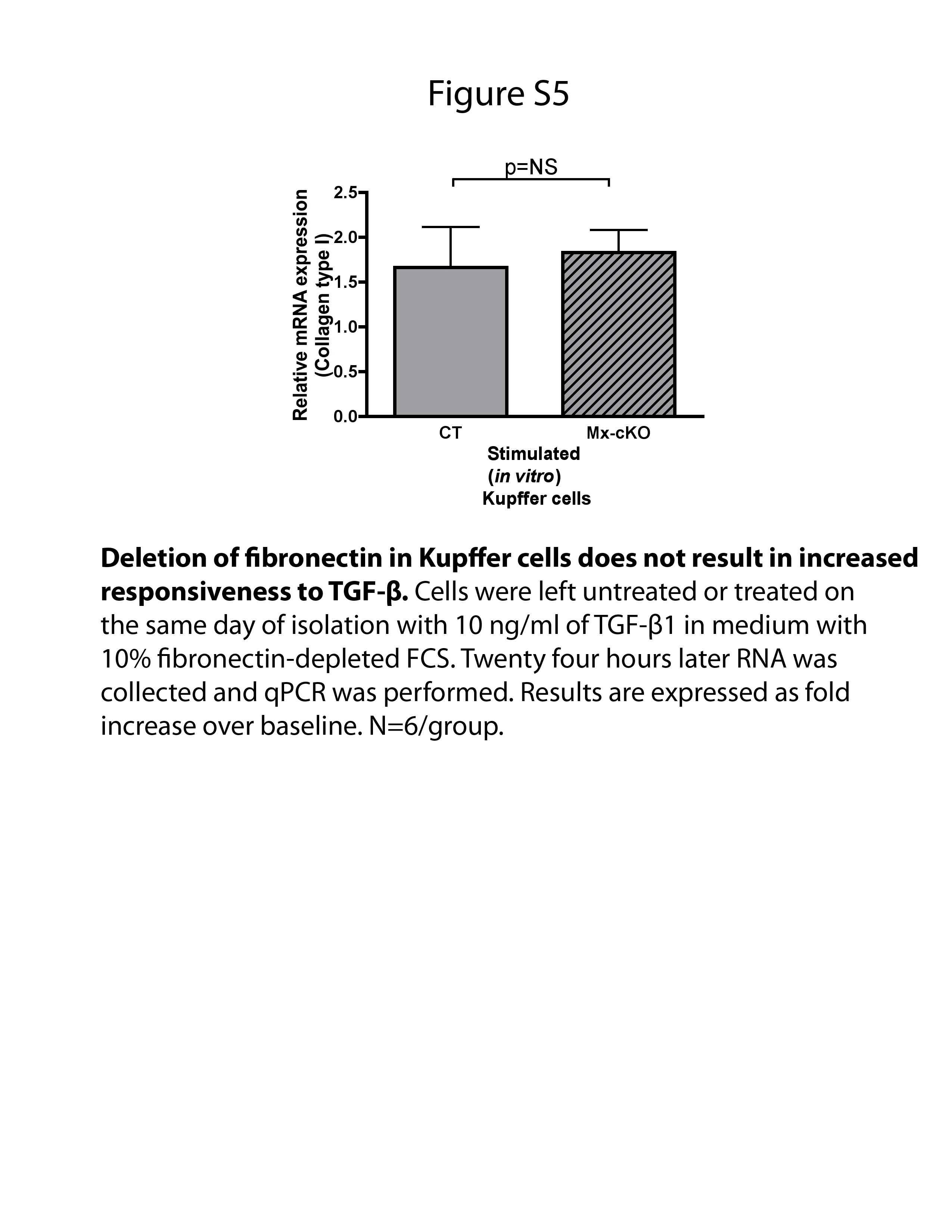

Supplement: Figure S5 — Deletion of fibronectin in Kupffer cells does not result in increased responsiveness to TGF-β. Cells were left untreated or treated on the same day of isolation with 10 ng/ml of TGF-β in medium with 10% fibronectin-depleted FCS. Twenty four hours later RNA was collected and qPCR was performed. Results are expressed as fold increase over baseline. N = 6/group. (TIF) [file pone.0028181.s005.tif]

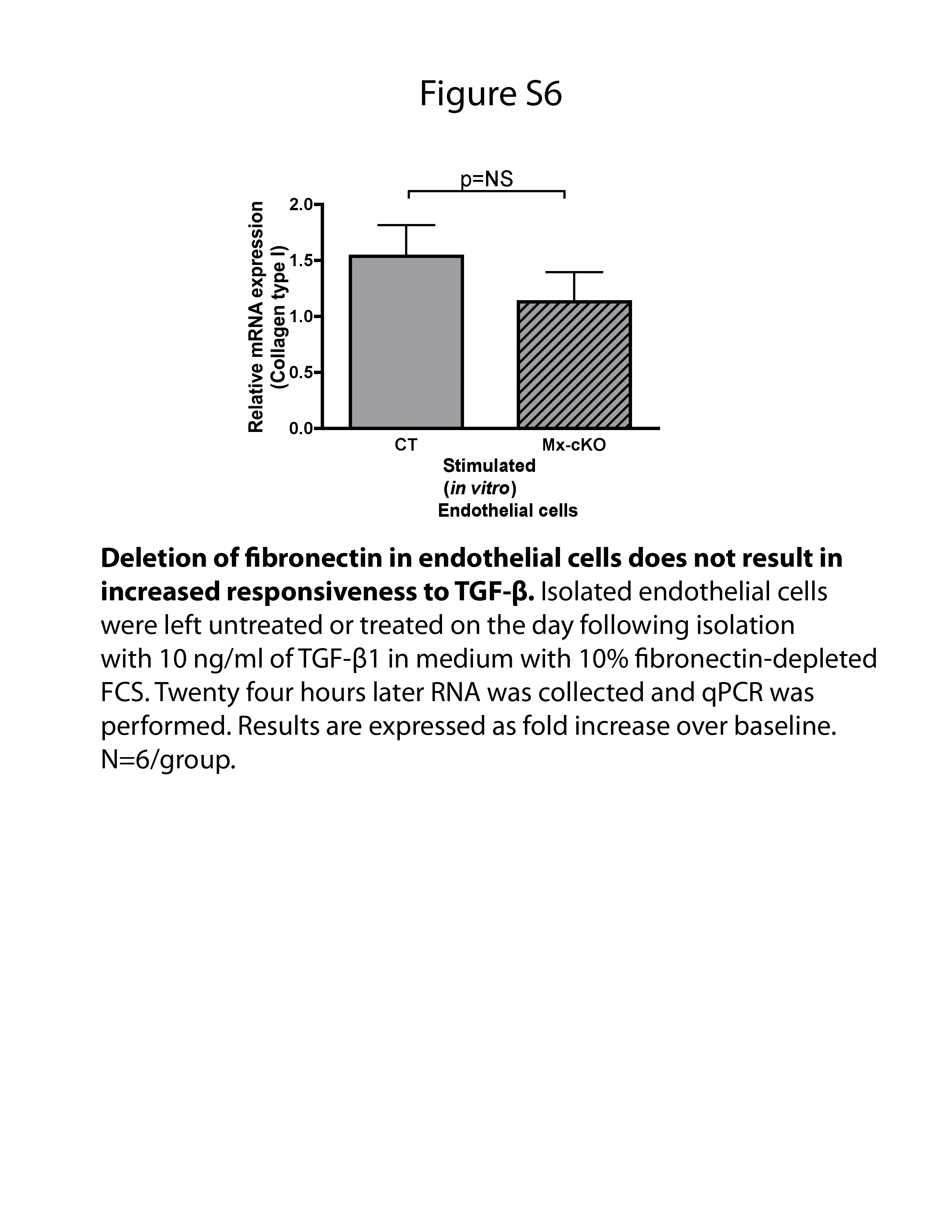

Supplement: Figure S6 — Deletion of fibronectin in endothelial cells does not result in increased responsiveness to TGF-β. Isolated endothelial cells were left untreated or treated on the day following isolation with 10 ng/ml of TGF-β in medium with 10% fibronectin-depleted FCS. Twenty four hours later RNA was collected and qPCR was performed. Results are expressed as fold increase over baseline. N = 6/group. (TIF) [file pone.0028181.s006.tif]

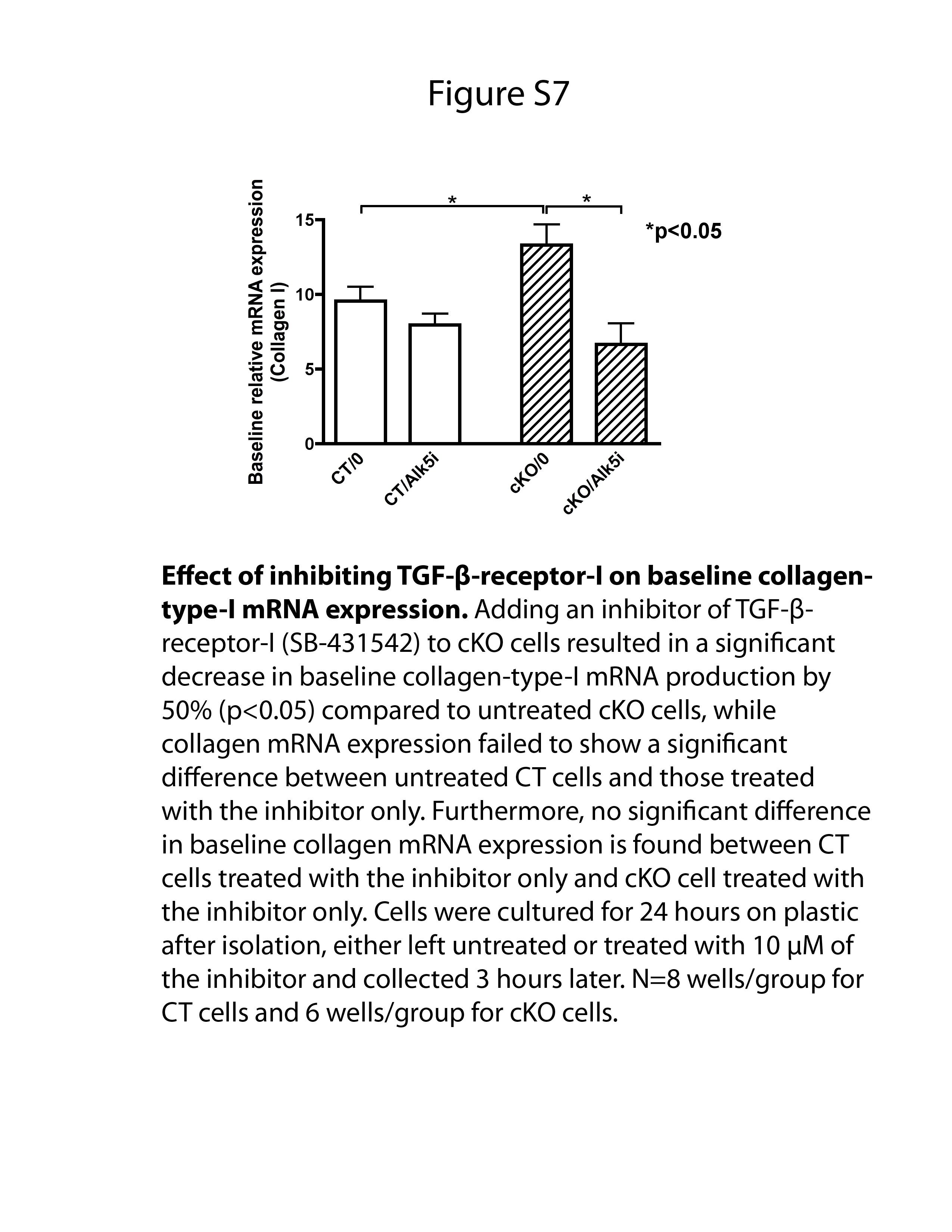

Supplement: Figure S7 — Effect of inhibiting TGF-β-receptor-I on baseline collagen-type-I mRNA expression. Adding an inhibitor of TGF-β-receptor-I (SB-431542) to cKO cells resulted in a significant decrease in baseline collagen-type-I mRNA production by 50% (p<0.05) compared to untreated cKO cells, while collagen mRNA expression failed to show a significant difference between untreated CT cells and those treated with the inhibitor only. Furthermore, no significant difference in baseline collagen mRNA expression is found between CT cells treated with the inhibitor only and cKO cell treated with the inhibitor only. Cells were cultured for 24 hours on plastic after isolation, either left untreated or treated with 10 µM of the inhibitor and collected 3 hours later. N = 8 wells/group for CT cells and 6 wells/group for cKO cells. (TIF) [file pone.0028181.s007.tif]

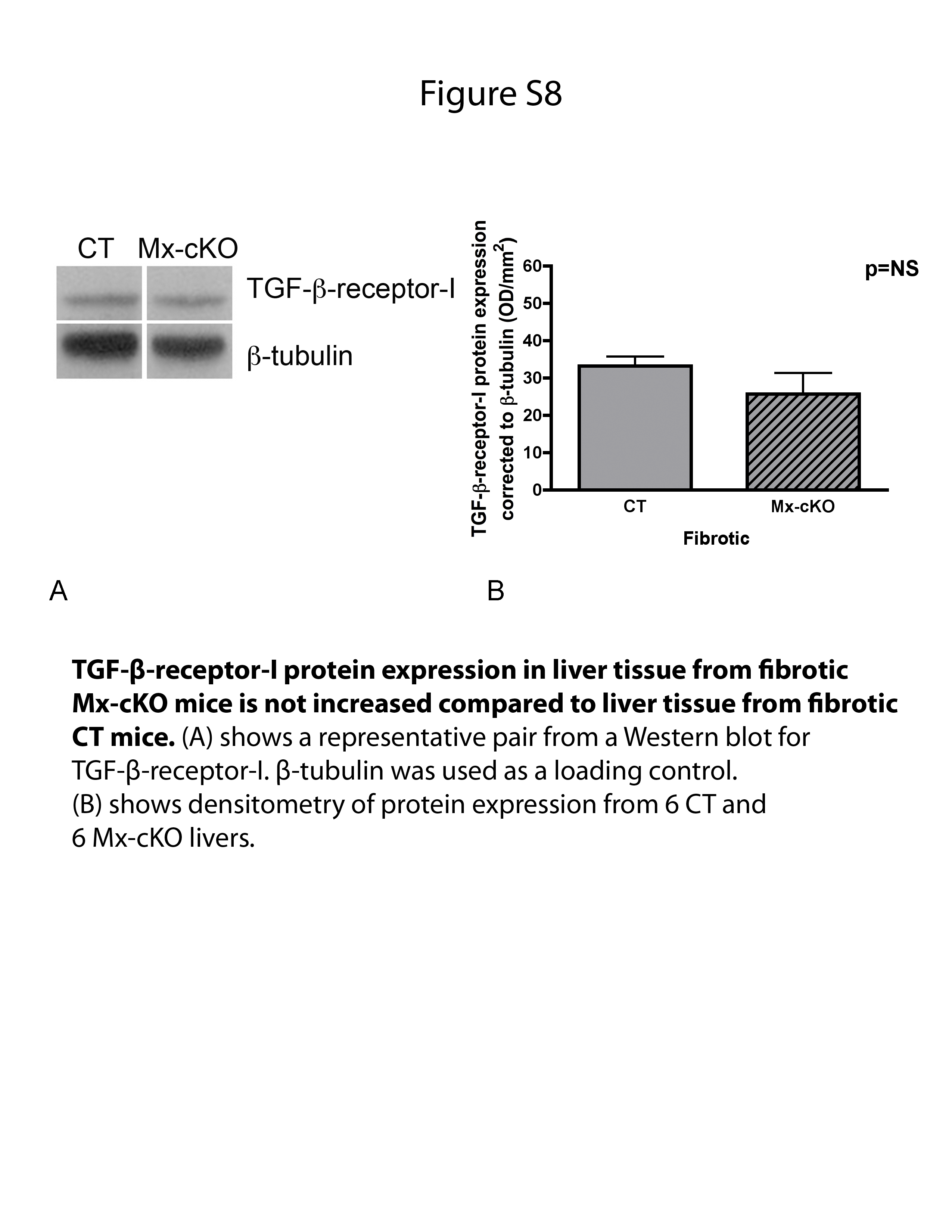

Supplement: Figure S8 — TGF-β-receptor-I protein expression in liver tissue from fibrotic Mx-cKO mice is not increased compared to liver tissue from fibrotic CT mice. (A) shows a representative pair from a Western blot for TGF-β-receptor-I. β-tubulin was used as a loading control. (B) shows densitometry of protein expression from 6 CT and 6 Mx-cKO livers. (TIF) [file pone.0028181.s008.tif]
